# Bifidobacterial biofilm formation is a multifactorial adaptive phenomenon in response to bile exposure

**DOI:** 10.1038/s41598-020-68179-9

**Published:** 2020-07-14

**Authors:** Sandra M. Kelly, Noreen Lanigan, Ian J. O’Neill, Francesca Bottacini, Gabriele Andrea Lugli, Alice Viappiani, Francesca Turroni, Marco Ventura, Douwe van Sinderen

**Affiliations:** 1 School of Microbiology, University College Cork, Western Road, Cork, Ireland; 2 APC Microbiome Ireland, University College Cork, Western Road, Cork, Ireland; 3 Laboratory of Probiogenomics, Department of Chemistry, Life Sciences, and Environmental Sustainability, University of Parma, Parma, Italy; 4 GenProbio Srl, 43124 Parma, Italy; 5 Microbiome Research Hub, University of Parma, Parma, Italy

**Keywords:** Bacteriology, Biofilms, Microbiome, Microbial ecology, Microbiology, Bacterial physiology

## Abstract

In the current study, we show that biofilm formation by various strains and species belonging to *Bifidobacterium*, a genus that includes gut commensals with reported health-promoting activities, is induced by high concentrations of bile (0.5% (w/v) or higher) and individual bile salts (20 mM or higher), rather than by acid or osmotic stress. The transcriptomic response of a bifidobacterial prototype *Bifidobacterium breve* UCC2003 to such high bile concentrations was investigated and a random transposon bank of *B. breve* UCC2003 was screened for mutants that affect biofilm formation in order to identify genes involved in this adaptive process. Eleven mutants affected in their ability to form a biofilm were identified, while biofilm formation capacity of an insertional mutation in *luxS* and an exopolysaccharide (EPS) negative *B*. *breve* UCC2003 was also studied. Reduced capacity to form biofilm also caused reduced viability when exposed to porcine bile. We propose that bifidobacterial biofilm formation is an adaptive response to high concentrations of bile in order to avoid bactericidal effects of high bile concentrations in the gastrointestinal environment. Biofilm formation appears to be a multi-factorial process involving EPS production, proteins and extracellular DNA release, representing a crucial strategy in response to bile stress in order to enhance fitness in the gut environment.

## Introduction

Biofilms are microbial communities that are attached to a surface and are enclosed/structured by an extracellular matrix^1^. Biofilms may also form when free floating cells clump together or adhere to particulate matter, this being referred to as microcolony formation^2^. Biofilm formation is a complex process involving multiple steps, including initial attachment, accumulation, maturation and dispersal^3^. Initial attachment is reversible and can be driven by electrostatic interactions; attachment to a surface can also be mediated by cell wall-associated proteins that bind to a substrate-covered surface or extracellular DNA (eDNA) release, where DNA released by cell lysis coats the surface and changes surface properties to allow attachment^4,5^. The accumulation phase of a biofilm can be mediated by cell wall associated protein interactions or extracellular matrix (EM) secretion^5,6^. The EM of biofilms may be composed of extracellular polysaccharide (EPS), DNA and/or proteins^7–9^. Finally, following maturation, individual cells may disperse from the biofilm to resume planktonic growth^10^. Biofilm formation is often triggered in response to environmental stresses, such as nutrient starvation, antibiotics, pH and bile, or induced through quorum sensing systems, such as the Agr or autoinducer-2 (AI-2) systems^11–14^.

Bifidobacteria are non-motile gut commensals, some of which are purported to exert health-promoting or probiotic properties (see review^15^). Particular bifidobacterial strains are for this reason included in certain ‘functional foods’ so as to bestow these benefits to the host that ingests them^16^. However, whether bifidobacteria colonise from birth or are ingested as a probiotic they will encounter and must overcome stresses in the gastrointestinal tract (GIT), such as low pH, bile, osmotic stress and nutrient starvation, as well as compete with other members of the microbiota^17^. Bile is present as a gradient along the GIT (40 mM to 0.5 mM), being highest in the small intestine and lowest in the colon^18,19^; however, bile/bile salt concentrations will vary greatly upon ingestion of (certain types of) food.

Bile and its constituent bile salts represent a major stress-inducing factor to bacteria in the GIT environment due to their bactericidal properties^19–21^. There are different types of bile salts since primary bile salts such as chenodeoxycholic acid or cholic acid can be conjugated with either a taurine or glycine before secretion. Primary bile salts can also be dehydroxylated by the gut microbiota to form secondary bile acids which can also be conjugated by taurine or glycine^20^. Bile salts are bactericidal and target and disrupt the bacterial cell membrane^20^. In bifidobacteria bile resistance mechanisms include efflux of bile salts by multi-drug transporters^22–24^, compositional changes of the cell membrane^25–27^, F_0_F_1_-ATPase proton efflux^28^, changes in metabolism^29–31^ and hydrolysis of bile salts^32,33^. Bile has previously been shown to induce biofilm formation in certain gut commensals, such as particular species/strains of *Bacteroides*, bifidobacteria and lactobacilli^34–36^. Therefore, it is important to study biofilm formation in commensal strains, such as bifidobacteria, and to obtain insights into how they adjust to and survive bile stress, and how this contributes to gut colonisation.

Bifidobacteria have previously been shown to form microcolonies on the gut mucosal surface and food particulates isolated from the gut^37,38^. Currently, little is known about the molecular mechanisms of biofilm formation in bifidobacteria. Bile and bile salts at relatively high concentrations (0.5% taurocholic acid and 5% porcine bile) have previously been found to induce biofilm formation in bifidobacteria^34^. In many bacterial species a specific quorum sensing signalling system is required for the induction of biofilm formation. For example, the AI-2 system involves LuxS, a S-ribosylhomocysteinase, producing AI-2, which is released extracellularly, and then sensed by the LuxP, LsrB or RbsB receptors of two component systems which in turn cause transcriptional induction of genes involved in eDNA release and polysaccharide production and other genes involved in biofilm formation^39–41^. Previously, AI-2 activity has been detected by several bifidobacterial species and strains, while in addition the over-expression of LuxS in *Bifidobacterium longum* subsp. *longum* NCC2705 has been linked to increased biofilm formation^42–44^. The exposure and growth of *Bifidobacterium breve* UCC2003 to bile and bile salts has also been shown to cause increased transcription of *luxS* which is a homolog of the previously studied *luxS* in *B. longum* subsp. *longum* NCC2705^24,42^. An insertion mutant in *luxS* in *B*. *breve* UCC2003 has previously been demonstrated to negatively affect gut colonisation ability in a mouse model^43^. However, the effect of a *luxS* mutation on biofilm formation in *B*. *breve* UCC2003 was not investigated. Besides these studies, essentially nothing is known about the molecular mechanisms of biofilm formation in bifidobacteria.

The aim of this study was to identify at what physiologically relevant concentrations of bile/bile salts biofilm formation is induced, and to identify genes involved in bifidobacterial biofilm formation. Our findings indicate that biofilm formation is a multi-factorial response to high concentrations of bile which is likely to be crucial for survival and colonisation of bifidobacteria within the gut environment.

## Results

### Biofilm induction in bifidobacteria

**Figure 1:**
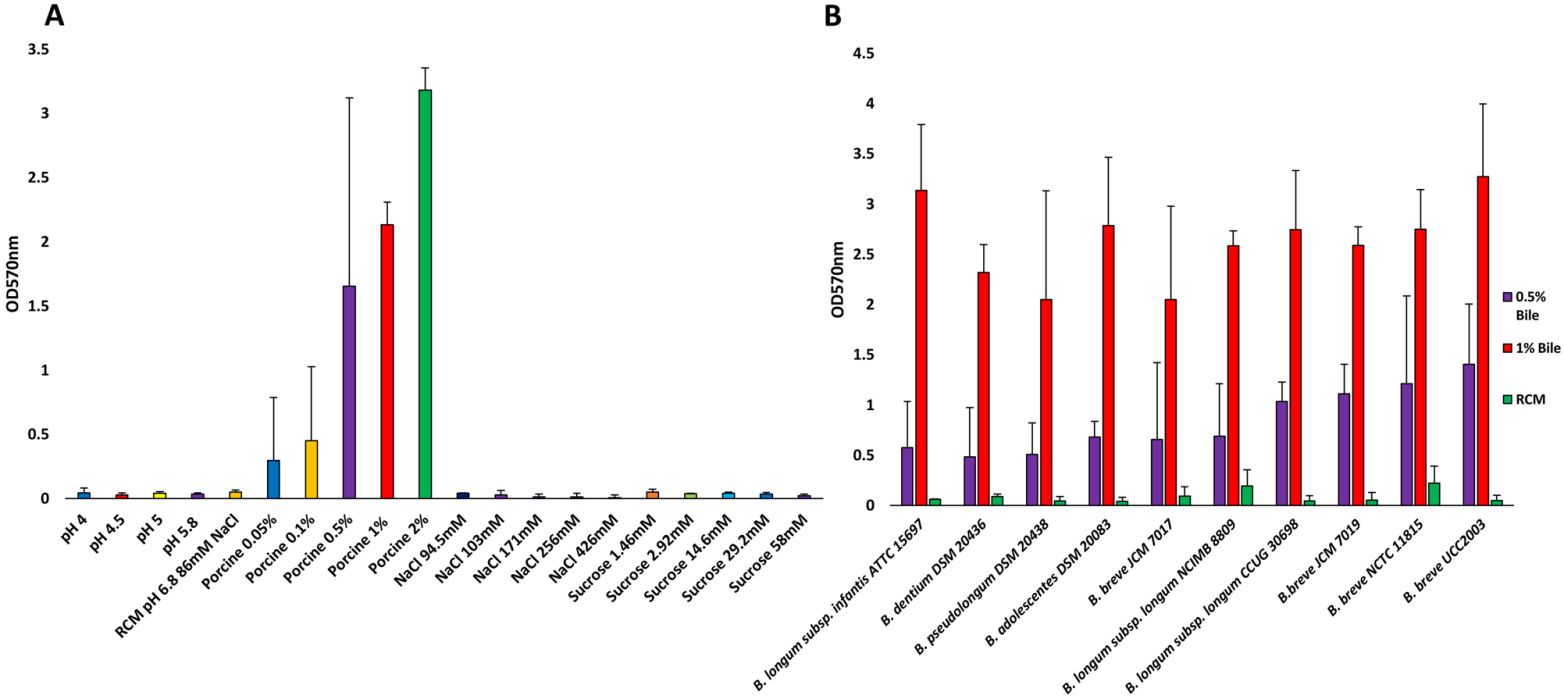
Biofilm formation by *Bifidobacterium breve* UCC2003 under different stress conditions (**A**). Biofilm was allowed to form for 24 h under various conditions including pH 4–6.8, sucrose 1.46 mM to 58 mM, NaCl 94.5 mM to 426 mM and porcine bile 0.05% to 2% (w/v). The pH of RCM was 6.8. Biofilm formation was assessed by crystal violet staining with absorbance read at O.D._570 nm_. Biofilm formation by several species/strains of bifidobacteria (**B**). Biofilm induced by addition of 0.5% or 1% (w/v) of porcine bile and allowed to form for 24 h. Biofilm was stained with crystal violet and the absorbance read at O.D._570 nm_. Negative controls with just RCM (non-inducing biofilm conditions) were also included for each species. Experiments were carried out in triplicate and error bars represent standard deviation.

Bifidobacteria may encounter various stresses in the GIT such as acid and bile salt stress^17^. In other bacterial species, acid stress^45^, salt stress^46^ and bile exposure are known to induce biofilm formation^47^. Bile salt (and by inference bile itself) concentrations vary along the GIT between 1 and 40 mM^21^. Therefore, we tested various conditions, using an established method for biofilm detection, the crystal violet assay, to investigate under what conditions biofilm formation occurs in bifidobacteria. Previously, biofilm formation by various bifidobacterial species had been detected by means of congo red and crystal violet staining assays, and shown to occur following exposure to 0.5% taurocholic acid and porcine bile at 5% (w/v)^34^. As expected, and using the prototype bifidobacterial gut commensal *B. breve* UCC2003 it was shown that biofilm formation indeed occurs following (porcine) bile exposure. However, because bile concentrations fluctuate throughout the GIT, we wanted to assess if biofilm formation is induced by other conditions pertinent to the intestinal environment and to what extent this occurs by varying porcine bile concentrations (Fig. 1). Our findings show that biofilm formation is not induced in *B. breve* UCC2003 by low pH or osmotic stress (NaCl or sucrose) as has been reported for other bacterial species^46^. All tested bile concentrations were considered physiologically relevant, and the biofilm-inducing effect of porcine bile was clearly shown to be dose dependent. Under the conditions tested biofilm formation by *B. breve* UCC2003 did not occur to any appreciable extent at bile concentrations of 0.05% and 0.1% (w/v), whereas at higher bile concentrations, i.e. 0.5% and above, clearly detectable biofilms were formed by this strain (Fig. 1A). Of note, addition of porcine bile to the RCM media did not cause a change in pH, and we therefore presume that the induction of biofilm formation is pH independent. Furthermore, we tested several bifidobacterial species/strains to assess if dose-dependent, bile-induced biofilm formation is exhibited by other members of the bifidobacterial genus. All examined bifidobacterial strains/species were indeed shown to produce a biofilm in the presence of 0.5% or 1% (w/v) porcine bile (Fig. 1B). Therefore, biofilm formation in the presence of high concentrations of bile seems to be a property elicited by multiple species/strains across the genus *Bifidobacterium*.

**Figure 2:**
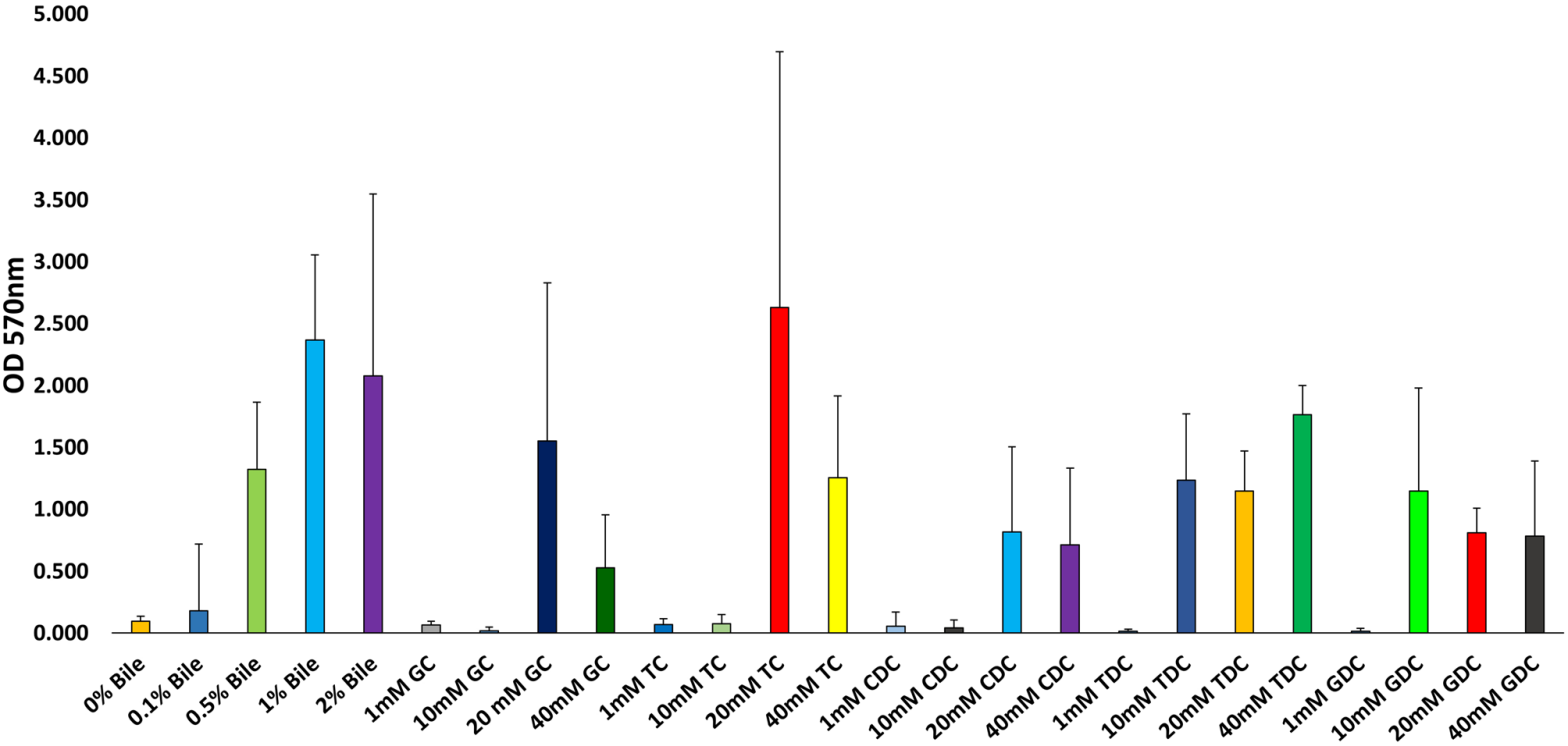
Biofilm formation of *Bifidobacterium breve* UCC2003 in response to bile salts. Biofilm formation was induced by addition of glycocholic acid (GC), taurocholic acid (TC), chenodeoxycholic acid (CDC), taurodeoxycholic acid (TDC) and glycodeoxycholic acid (GDC) at concentrations of 1 mM, 10 mM, 20 mM and 40 mM. Biofilm was allowed to form for 24 h, was staining using crystal violet and the absorbance read at O.D._570 nm_. Experiments were carried out in triplicate and error bars represent standard error of the mean.

Bile is a heterogeneous mix of various components including cholesterol, bile salts, proteins and bilirubin^20^. We therefore wanted to assess if bile salts alone are capable of inducing biofilm formation. Both taurine and glycine primary bile salts were tested along with their dehydroxy derivatives to see if any particular type of bile salt acts as a specific inducer for this process. Using *B. breve* UCC2003 it was shown that biofilm formation was triggered by individual bile salts (Fig. 2) and that biofilm formation typically occurs at higher concentrations of bile salts, i.e. 20 mM and 40 mM, while generally at lower concentrations, i.e. 1 mM and 10 mM, little or no biofilm was observed. Both trihydroxy-conjugated bile salts, taurocholic acid (TC) and glycocholic acid (GC), and dihydroxy-conjugated bile salts such as taurodeoxycholic acid (TDC), chenodeoxycholic acid (CDC) or glycodeoxycholic acid (GDC) induced biofilm formation (Fig. 2). Therefore, biofilm formation by bifidobacteria upon exposure to bile/bile salts is a common phenomenon and may represent an adaptation mechanism to specifically survive exposure to high levels of bile encountered in the GIT.

### Transcriptomic response of *Bifidobacterium breve* UCC2003 to a high concentration of bile

In order to determine the transcriptomic response of *B*. *breve* UCC2003 to a high concentration of bile and to assess whether these genes were implicated in biofilm formation (see below), this strain was cultivated to a corresponding O.D._600 nm_ value between 0.5 and 0.6, and then exposed for twenty minutes to 0.5% ((w/v) final concentration) porcine bile. Genes exhibiting transcriptional upregulation/downregulation following bile exposure, with an associated *p*-value of less than 0.05, are summarised in Table 1.

**Table 1:** Genes transcriptionally upregulated or downregulated in response to 0.5% (w/v) porcine bile.

Locus tag	Putative Function	Fold change in expression (Up regulation)	P-value
Bbr_0376	Hypothetical protein	10.08	9.18E−14
Bbr_1594	PTS system fructose/glucose (*fruA*)	6.09	4.99E−15
Bbr_0925	Permease MFS superfamily	5.49	0.000125407
Bbr_0204	Multi-domain protein fatty acids or polyketide synthesis	4.33	1.79E−10
Bbr_1558	Permease of ABC transporter	4.16	1.98E−06
Bbr_0205	Multi-domain protein fatty acids or polyketide synthesis	3.81	2.43E−08
Bbr_0521	Solute binding protein of ABC transporter, bac 3 family	3.76	1.65E−07
Bbr_1890	ATP binding protein for ABC transporter	3.67	4.95E−09
Bbr_0492	Galactokinase	3.52	0.004216372
Bbr_0188	Formate – tetrahydrofolate ligase	3.00	0.000253137
Bbr_1719	Type I Multi-functional Fatty Acid Synthase	2.78	1.07E−07
Bbr_1615	DNA- directed RNA Polymerase Alpha Chain	2.38	5.34E−06
Bbr_1010	HIT Family Hydrolase	2.35	0.004013209
Bbr_1638	RplB, 50S ribosomal L2 protein	2.05	3.04E−05
Bbr_0183	Guanine-hypoxanthine permease	2.04	8.92E−05
Bbr_0911	IscU – Iron sulfur scaffold protein	1.93	0.000509005
Bbr_1002	Transaldolase	1.87	8.14E−07
Bbr_0499	DNA-directed RNA polymerase beta' chain	1.79	1.65E−05
Bbr_0969	Homocysteine methyltransferase	1.67	0.000468744
Bbr_0377	Oxidoreductase aldo/keto reductase family	1.63	0.003458674
Bbr_0771	Acetate kinase	1.52	0.000906418
Bbr_0329	ATP synthase beta chain	1.42	0.000860229
Bbr_0328	ATP synthase gamma chain	1.41	0.000351287
Bbr_1202	Oligopeptide transport ATP-binding protein oppD	1.28	0.001120451
Bbr_0371	Polyribonucleotide nucleotidyltransferase	1.23	0.000351287
Bbr_0776	Xylulose-5-phosphate/Fructose-6-phosphate phosphoketolase	1.13	0.00154578

Locus tag	Putative function	Fold change in expression (downregulation)	P-value
Bbr_0579	Solute binding protein of ABC transporter system, iron siderophore, metallic cations (Zn/Mn transport)	31.39	4.25E−39
Bbr_0808	ATP-binding protein of ABC transporter system	30.33	4.51E−15
Bbr_0538	Cysteine synthase	12.64	2.48E−06
Bbr_1354	Transciptional regulator	12.16	0.004471376
Bbr_0849	NagC/XylR-type transciptional regulator	11.58	2.80E−08
Bbr_0008	Transcriptional regulator LacI family	11.01	0.002749448
Bbr_1248	Glucosamine-6-phosphate isomerase	9.41	0.000796378
Bbr_1860	Solute binding protein of ABC transporter system for sugars	9.15	0.004306336
Bbr_0083	(Filamentation induced by cAMP) Fic family protein	9.04	0.00351993
Bbr_1791	Phosphoglycerol transferase	7.13	5.68E−05
Bbr_1781	ClpB protein	6.59	3.08E−07
Bbr_1506	Cyclopropane-fatty-acyl-phospholipid synthase	5.84	2.74E−05
Bbr_1793	ATP-binding protein ABC transporter system for polysaccharides	4.55	0.000502173
Bbr_0751	Solute-binding protein of ABC transporter system for metals	4.42	0.000817759
Bbr_1590	Solute-binding protein of ABC transporter system for sugars	4.26	3.50E-08
Bbr_0106	Cellodextrin binding protein	4.17	0.000211367
Bbr_0348	Aspartate ammonia-lyase	4.06	0.005724464
Bbr_0070	Cell division protein FtsW	3.89	0.003286088
Bbr_1251	N-acetylglucosamine repressor	3.78	0.005702581
Bbr_0417	Solute-binding protein of ABC transporter system for sugars galactan metabolism	3.37	4.61E−05
Bbr_1790	Phosphoglycerol transferase	3.26	0.003699621
Bbr_0027	Permease protein of ABC transporter system for sugars	3.17	0.000263903
Bbr_0809	Permease protein of ABC transporter system	2.15	0.005541407
Bbr_1889	Cell surface protein with gram positive anchor domain	1.98	0.005000633
Bbr_0746	14-alpha-glucan branching enzyme	1.82	0.002004982
Bbr_1574	Phosphoglycerate mutase	1.79	0.00071798
Bbr_1710	Ribokinase	1.75	0.001314349

All fold changes are p < 0.05.

Various genes predicted to be involved in transport and metabolism of carbohydrates were significantly upregulated following 0.5% bile exposure. Transcription of a gene encoding a putative PEP-PTS system (Bbr_1594), which previously was shown to be induced by glucose^48^, was upregulated sixfold under the imposed bile exposure conditions. Similarly, genes predicted to encode an ABC-type transporter permease (Bbr_1558), an ATP-binding protein for an ABC-type transporter (Bbr_1890), galactokinase (Bbr_0492), acetate kinase (Bbr_0771) and xylulose-5-phosphate/Fructose-6-phosphate phosphoketolase (Bbr_0776) were shown to be transcriptionally upregulated under these conditions, indicating that carbohydrate uptake and active carbohydrate metabolism are associated with the adaptive response to bile stress. However, our results also show that transcription of other genes involved in transport and metabolism of carbohydrates was subject to downregulation upon exposure to bile. These included an ATP-binding protein of an ABC-type transporter system (Bbr_0808), a glucosamine 6-phosphate isomerase (Bbr_1248), a cellodextrin binding protein (Bbr_0106), 1–4 α glucan branching enzyme and others summarised in Table 1. Therefore, it seems that a specific response of increased carbohydrate metabolism is induced following the imposition of bile stress.

A solute binding protein (SBP) of an ABC-type transporter (Bbr_0521) of the bac3 family possibly involved with glutamate and histidine uptake was also downregulated. A presumed SBP (Bbr_0579) implicated involved in Zn/Mn transport and previously found to be upregulated under iron limitation conditions^49^, was downregulated 31 fold. Transcription of genes predicted to be involved in polyketide synthesis (Bbr_0204/0205)/ fatty acid metabolism (Bbr_1719) also incur upregulation in response to bile shock. Other genes, whose transcription was shown to increase upon bile exposure, were predicted to be involved in cysteine metabolism (Bbr_0969), ATP production (Bbr_328/329), iron-sulfur metabolism (Bbr_0911) and an ATP component of the oligopeptide nucleotide transporter OppD (Bbr_1202).

Whether or not the genes involved in bile resistance and genes involved in biofilm formation are interconnected remains to be seen. Therefore, we decided to investigate which genes are involved in biofilm formation and to determine if these genes are akin to the genes upregulated in the shock exposure to bile.

### Screening of a transposon-mediated insertion mutant library of *B*. *breve* UCC2003

In order to identify genes involved in biofilm formation, a previously described transposon mutant library of *B*. *breve* UCC2003^49,50^ was screened for mutants affected in biofilm formation. Biofilm induction was achieved employing exposure of individual mutants to high concentrations of porcine bile, 0.5 or 1% (w/v), for 24 h; biofilm biomass was stained using crystal violet, solubilised in acetic acid and an associated O.D._570 nm_ measurement was taken to perform a semi-quantitative assessment of biofilm biomass. A reduced O.D._570 nm_ value (compared to that obtained for the wild type strain *B. breve* UCC2003) indicated a reduction in biofilm biomass formation and suggested that the transposon had mutated a gene involved in biofilm formation. A positive control of *B*. *breve* UCC2003 and transposon mutants grown in RCM was also included to exclude mutants that were simply impaired in growth (O.D._600nm_ value being < 0.5) which could have reduced biofilm biomass because of reduced cell numbers due to poor growth. The screen was carried out with RCM to prevent identifying mutants defective in growth of a single carbon source (as RCM contains both glucose and starch). Transposon mutants identified in the primary screen where retested in a confirmatory screen in triplicate to ensure no false positives were isolated. 10,000 transposon mutants were screened from the *B. breve* UCC2003-derived transposon library, resulting in the identification of eleven mutants that were shown to be clearly and consistently affected in their ability to form a biofilm (Table 2; Supplementary Figure S1).

**Table 2:** Transposon insertions isolated in crystal violet biofilm screen.

Mutant	Gene locus	Function
Bbr_1202*	*oppD2/oppB2/oppC1*operon	Oligopeptide transporter
Bbr_1738	*dapE*	Succinyl-diaminopimelate desuccinylase, lysine and cell wall synthesis
Bbr_1901	*nrdH*, *nrdI*, *nrdE* operon	Ribonucleotide reductase
Bbr_0074	*pepX*	Xaa –Prolyl Peptidase
Bbr_1719/20/21^†^	*accC/accD/fas* operon	Fatty acid biosynthesis
Bbr_200	NADH Flavin reductase	DNA binding protein/NADH Flavin reductase
Bbr_201	DNA binding protein	DNA binding protein/AAA ATPase
Bbr_1654/53/52/51	*serA2*	Non-functional conserved protein/Phosphoglycerate dehydrogenase/Thymidlate synthase
Bbr_0060	*glgP1*	Glycogen phosphorylase
Bbr_1353	*proP*	Osmolarity/stress MFS
Bbr_1580	Conserved Hypothetical Protein	Transmembrane protein/hydrolase

*Gene was isolated twice in mutant screen. (Distinct mutants in the same gene).

^†^Gene was isolated three times in mutant screen. (Distinct mutants in the same gene).

**Figure 3:**
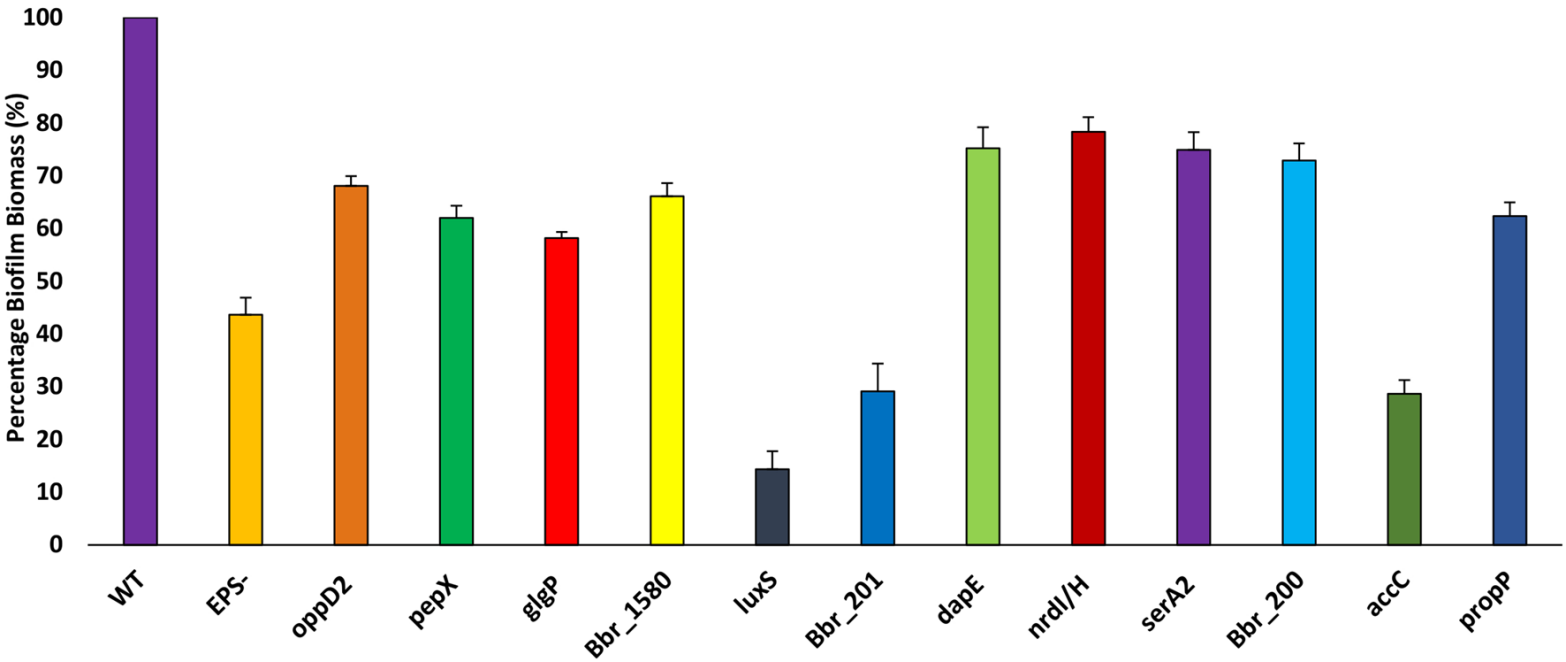
Biofilm formation by mutants screened from a *Bifidobacterium breve* UCC2003 transposon mutant bank. A transposon mutant bank was screened using the crystal violet assay. Biofilm was induced with 0.5% (w/v) porcine bile and allowed to form for 24 h. An insertional mutant *B*. *breve* UCC2003 *luxS*, and EPS deficient strain, *B. breve* UCC2003 ${\text{EPS}}^{-}$, were also screened. A mutant in biofilm formation was assumed to have reduced biofilm biomass, as compared to the wildtype, due to reduced absorbance at O.D._570 nm_. Experiments were carried out in triplicate and error bars represent standard error of the mean.

The location of the transposon in individual mutants was identified by direct inverse PCR (iPCR) amplification or arbitrary primed PCR and subsequent sequencing (see “Materials and Methods”) and predicted gene functions were assigned by BlastP analysis. Alongside these mutants two other previously described mutants in a gene encoding a predicted priming glycosyl hydrolase causing loss of EPS production (EPS^−^) and a mutant in the gene for the AI-2-producing LuxS enzyme were also tested^43,51^. All eleven identified transposon and the two additionally selected mutants tested exhibited reduced biofilm biomass compared to *B*. *breve* UCC2003 WT at 24 h as determined by the crystal violet assay (Fig. 3). The *B*. *breve* UCC2003 EPS^−^ mutant was shown to elicit substantially reduced biofilm biomass as compared to the wildtype suggesting that EPS is important in biofilm formation. Several genes involved in metabolism and physiology where found to be involved in biofilm formation, such as (i) *nrdHIE*, which encodes a ribonucleotide reductase, (ii) SerA2, a phosphoglycerate dehydrogenase/ thymidlate synthesis, (iii) Bbr_200, an NADH flavin reductase, (iv) Bbr_201, an AAA ATPase, and (v) *glgP1*, a glycogen phosphorylase, which is an enzyme responsible for the breakdown of glycogen^52^. Transposon-mediated disruption of genes that influence the composition and properties of the cell wall envelope also had an impact on biofilm formation, such as *dapE*. DapE is a *N*-succinyl-l,l-diaminopimelic acid desuccinylase part of the lysine/*meso*-diaminopimelate (mDAP) pathway that produces lysine for protein synthesis and both lysine and mDAP are required for peptidoglycan synthesis^53^. A mutant in a gene responsible for type I fatty acid biosynthesis (*fas*) also exhibited reduced biofilm biomass. A total of three transposon mutants in *accC* were isolated from the transposon bank screen. The *accC*, *accD* and *fas* genes, putatively encoding the acetyl Co-A α chain, acetyl Co-A β chain and the fatty acid synthase enzymes, respectively, are adjacent to each other and mutations in these genes are believed to interfere with fatty acid biosynthesis. Furthermore, mutations in genes involved in amino acid metabolism, such as a predicted oligopeptide transporter OppD2 and a predicted peptidase PepX, were shown to affect biofilm formation.

From the above mutant screen, it is apparent that biofilm formation is a complex process involving a diverse set of genes involved in, among others, EPS production, in S-ribosylhomocysteinase production, as well as carbon, fatty acid and peptide metabolism. Some of the genes, such as Bbr_1719; involved in fatty acid synthesis (*accC*), and Bbr_1202 (*oppD2*) found in the screen were also upregulated in response to high concentrations of bile. Therefore, we wondered if biofilm was a survival strategy in response to high concentrations of bile.

### Biofilm viability

**Figure 4:**
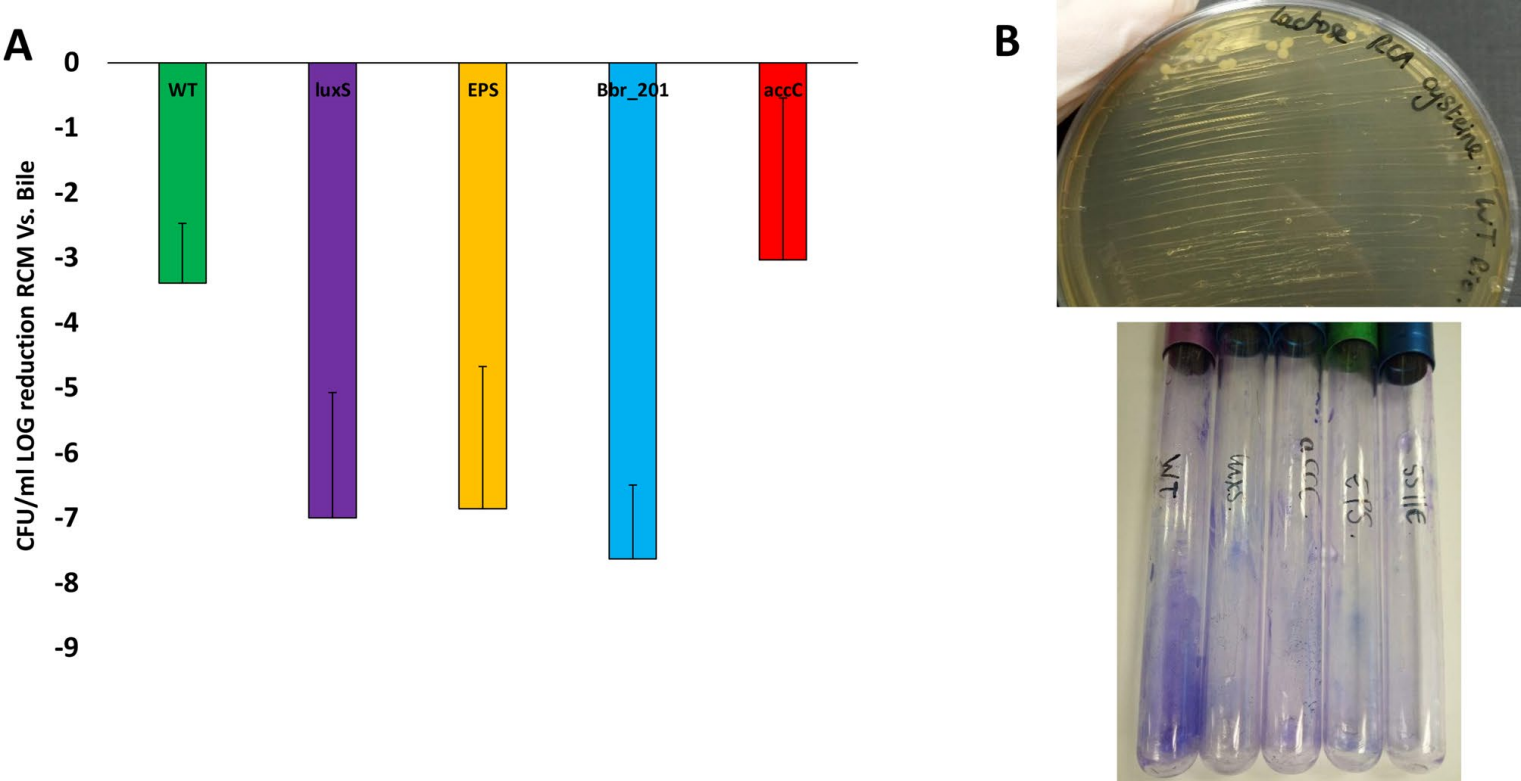
Viability of Bifidobacteria after 24 h growth in porcine bile. *B.* *breve* UCC2003 WT, *B*. *breve* UCC2003 *luxS*, *B. breve* UCC2003 ${\text{EPS}}^{-}$*, B. breve* UCC2003 *accC* and *B .breve* UCC2003 Bbr_201 were grown in a microtiter plate in RCM supplemented with 0.5% (w/v) of porcine bile (biofilm formation conditions) and incubated for 24 h. Culture media was then diluted and spot plated on RCA to see if viable bacteria could be recovered and the CFU/ml was calculated (**A**). Experiments were carried out in triplicate and error bars represent standard deviation. Biofilm was also induced in testubes by growing the above strains in RCM supplemented with 0.5% (w/v) of porcine bile and incubated for 24 h (**B**). Supernant was removed and test tubes were washed twice to remove planktonic cells. Biofilm was then scraped off the test tubes, where formed, with a pipette tip and streaked out on RCA supplemeted with lactose and cysteine to obtain viable colony counts (top image). Test tubes were also stained with crystal violet to visualise biofilm (bottom image).

Biofilm formation seems to be associated with bile resistance and in order to investigate if this biofilm forming ability is positively correlated with enhanced survival following bile exposure, *B*. *breve* UCC2003 WT, the *luxS* insertion mutant, the EPS^−^ mutant, as well as transposon mutants in *accC* and Bbr_201 were grown for 24 h in RCM supplemented with (0.5%; w/v) or without porcine bile. Culture media was then diluted in PBS and spot plated on RCA to determine viable counts. Under these conditions *B*. *breve* UCC2003 WT and *accC* mutant were shown to exhibit the highest survival level compared to any of the other mutants (Fig. 4A). To test if the biofilm formed was viable after 24Hrs, these strains were also grown in test tubes in the presence of porcine bile (0.5%; w/v) and left for 24 h to allow biofilm to form. Biofilm was then scraped off with a pipette tip and restreaked on RCA supplemented with cysteine and 0.5% lactose (Fig. 4B). The RCA plates were then incubated for 48 h and any colonies present counted. Viable colonies could be recovered from biofilm of *B. breve* UCC2003 WT and for all the mutants even though these mutants had less biofilm biomass. Therefore, these results suggest that the biofilm biomass itself is viable and that biofilm formation can increase resistance to high concentrations of bile.

### Biofilm matrix composition

**Figure 5:**
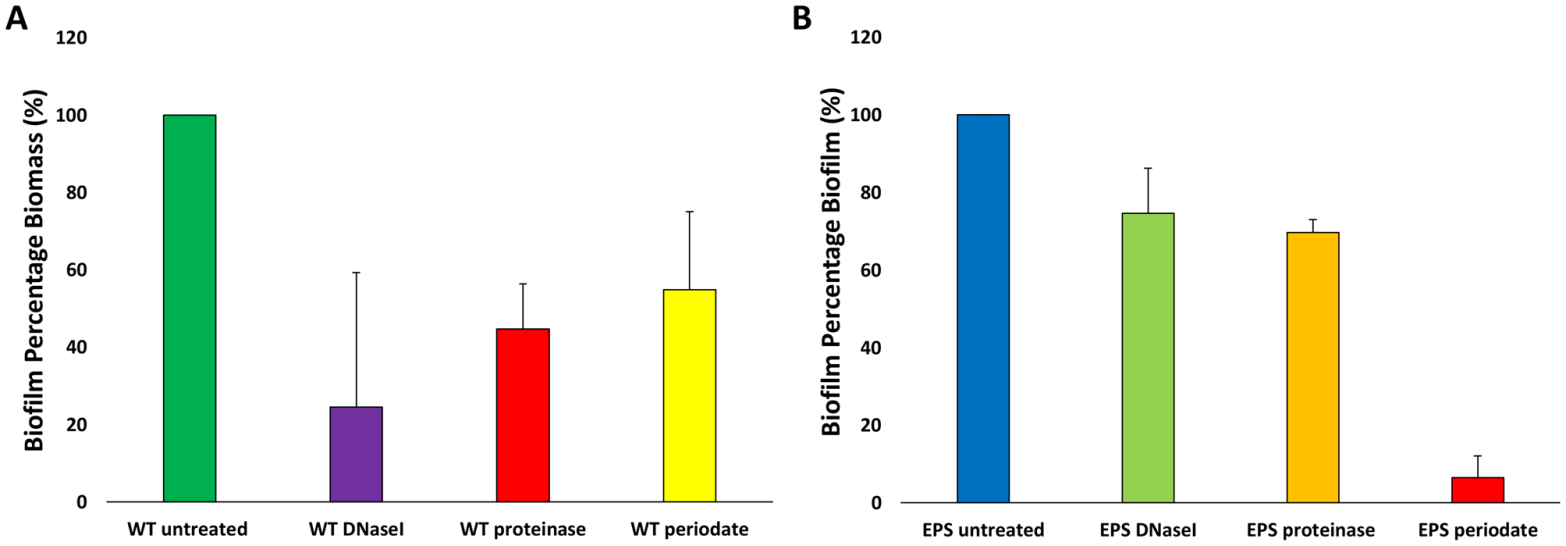
Inhibition of biofilm attachment of *Bifidobacterium breve* UCC2003 wildtype (WT) (**A**) and *Bifidobacterium breve* UCC2003 exopolysaccharide deficient (EPS^−^) (**B**). Biofilm was induced by supplemmenting media with 0.5% (w/v) porcine bile and was additionally incubated with DNaseI (10 U/ml), proteinase (0.95 mg/ml) or sodium (meta)periodate (4 mM) or left untreated. Biofilms were then left to form for 24 h, biofilm biomass was stained with crystal violet and absorbance read at O.D._570 nm_. Maximal biofilm production was taken to be 100% for *B*. *breve* UCC2003 WT and *B. breve* UCC2003 EPS^−^ when comparing effects of DNaseI, proteinase and sodium (meta) periodate on these individual strains. All experiments were carried out in triplicate and errors bars represent standard deviations.

In other bacterial species, cell wall associated proteins, EPS and eDNA are involved in the initiation and accumulation stages of biofilm^46,54,55^. Therefore, in order to get an insight into the initiation stages of biofilm formation, biofilms for *B*. *breve* UCC2003 wildtype were set up in microtiter plates as above but were also incubated with proteinase K, to degrade proteins, or DNaseI, to degrade eDNA, and sodium metaperiodate, to oxidise EPS/cell surface carbohydrates, in order to assess if proteins, eDNA or extracellular surface carbohydrates play a role in (the initial stages of) biofilm formation (Fig. 5). Incubation with proteinase K, DNaseI and sodium (meta) periodate was shown to cause a reduced biofilm biomass after 24 h as indicated as a reduced O.D._570 nm_ value as compared to untreated *B*. *breve* UCC2003 WT (Fig. 5A) biofilm suggesting that the attachment and accumulation phases are mediated by a combination of proteins, extracellular DNA release and carbohydrate secretion, presumably EPS mediated. This indicates that macromolecules such as cell wall-associated proteins, eDNA and EPS are involved in the initial attachment and accumulation phases of bifidobacterial biofilm formation. The *B*. *breve* UCC2003 EPS-negative mutant appeared to produce substantially less biofilm biomass than the WT, and extended treatment with DNaseI and proteinase was shown to reduce biofilm yet did not abolish biofilm completely. Treatment with sodium (meta) periodate did substantially reduce biofilm formation suggesting perhaps other cell wall-associated polysaccharides are important in biofilm formation. In the latter context it is relevant to note that *B*. *breve* UCC2003 has been reported to contain two EPS clusters^51,56^.

**Figure 6:**
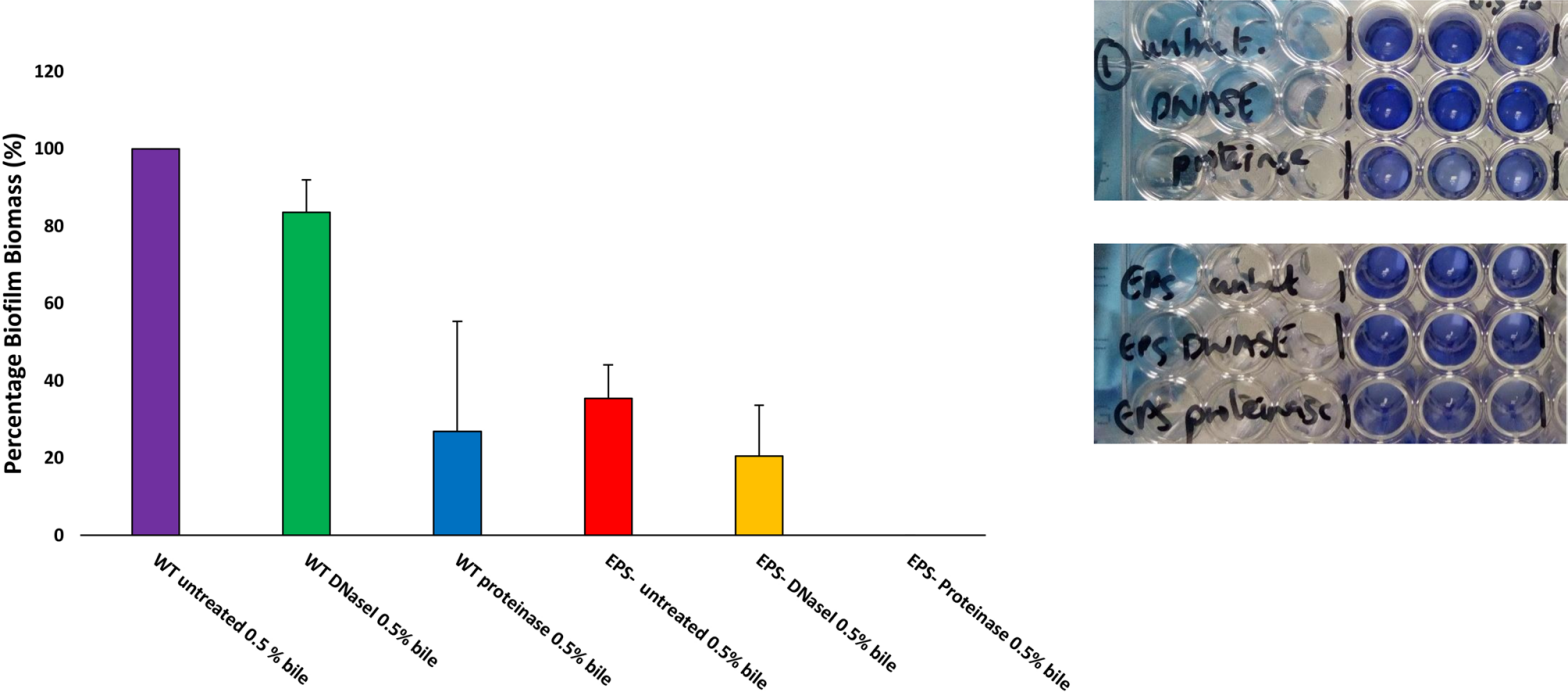
Dispersal of mature biofilms of *Bifidobacterium breve* UCC2003 wildtype (WT) and a *B. breve* UCC2003 derivative deficient in exopolysaccharide production (EPS^−^). Biofilm was induced by supplementation of media with 0.5% (w/v) porcine bile and biofilms were allowed to form for 24 h. Mature biofilms were then treated with DNaseI (10 U/ml) in 5 mM MgCl_2_ and 0.95 mg/ml proteinase K in 20 mM in Tris–HCl. Biofilms were stained with crystal violet and absorbance read at ${\text{O.D.}}_{\text{570nm}}$. Biofilm formation of *B*. *breve* UCC2003 WT was taken to be 100%. All experiments were carried out in triplicate and error bars represent standard deviations.

To investigate the composition of the EM of mature biofilms of the *B*. *breve* UCC2003 WT formed after 24 h, biofilms were enzymatically treated with proteinase K and DNaseI to determine if protein and/or DNA contributed to the EM, respectively **(**Fig. 6). Proteinase K was able to disperse mature biofilm of *B*. *breve* UCC2003 WT, whereas DNaseI could not. This suggests that while extracellular DNA release may be important in the initial stages of biofilm formation it may not be as important in established mature biofilm structures. Proteinase K could also not completely disperse biofilm in *B. breve* UCC2003 WT, suggesting that mature biofilm composition is a multifactorial process, involving multiple macromolecules. In fact, complete (mature) biofilm dispersal was only observed when the *B*. *breve* UCC2003 EPS^−^ mutant was treated with proteinase K. This suggests that both EPS and proteins play an important role in mature biofilm formation.

## Discussion

Bifidobacteria are gut commensals and to survive in the GIT environment they must be able to survive bile exposure. Our findings show that bifidobacteria form a biofilm following exposure to high concentrations of porcine bile. Porcine bile possesses a glycine:taurine ratio which is similar to that of human bile^57^. Previous studies characterising the bifidobacterial bile response used bovine bile, rather than porcine bile, while also employing bile/bile salts at lower concentrations than those shown to induce biofilm formation^24, 25,28,29^. It is important to assess the bifidobacterial response to various concentrations of bile as there is a gradient of bile in the GIT. The transcriptomic response of *B*. *breve* UCC2003 to a high concentration (i.e. 0.5% w/v or higher) of porcine bile was also distinct from bile exposure to lower concentrations of bile, oxgall 0.15% (w/v) and cholate 0.06% (w/v), as previously reported^24^. The transcriptomic response of *B*. *breve* UCC2003 to a high concentration of bile was shown to involve specific response in carbohydrate metabolism. This is in agreement with previous proteomic studies assessing bile response, where the expression of glycolytic enzymes and pyruvate catabolism enzymes, such as acetate kinase and xylulose-5-phosphate/fructose-6-phosphate phosphoketolase, was upregulated^29,30,58^. Bile-adapted bifidobacterial strains have a different carbohydrate preference as compared to WT strains^59^. Therefore, bile shock seems to invoke specific changes in carbohydrate uptake, storage and metabolism that may be important to survive high bile concentrations.

Genes involved in bile resistance also seem to be connected to biofilm formation. Our findings show that on exposure to high concentrations of bile fatty acid biosynthesis is induced, which also contributed to biofilm formation. A mutant in *accC* was shown to elicit increased resistance to bile, which suggests that fatty acid synthesis is not only important for biofilm formation but also for bile resistance. Previous studies have reported that transcription of the fatty acid synthase genes is downregulated when bifidobacteria are exposed to bile^24,27^. However, these studies were conducted at lower concentrations of bile with either bovine bile and/or individual bile salts rather than porcine bile and this may explain this apparent discrepancy. It is unknown why fatty acid metabolism is important in bile resistance. It has previously been shown that bile induces biofilm formation due to its capacity to increase surface hydrophobicity of bifidobacterial cells^34^. Therefore, changes in surface hydrophobicity and perhaps membrane permeability due to altered fatty acid synthesis may help to resist the bacteriocidal effects of bile. Similarly, OppD2 was shown to be upregulated and involved in biofilm formation. It has previously been reported that OppA production is upregulated in bifidobacteria upon exposure to bile and shown to allow increased uptake of oligopeptides^24,60^. Oligopeptide transporters have also been implicated in bile resistance in *Lactobacillus salivarius*^61^, although the precise manner by which peptides are involved in biofilm formation and bile resistance is currently not clear.

Bifidobacteria have been shown to form biofilm in the GIT environment^37,38^. We identified various genes involved in biofilm formation and we have shown that some of the corresponding mutants exhibit reduced viability following growth in bile. The *luxS* mutant was previously shown to impact on GIT colonisation in a mouse model^43^. Similarly, insertion in *luxS* has an impact on biofilm formation and colonisation persistence in lactobacilli^62^. However, the effect of a *luxS* insertion was not found to be exclusively due to absence of AI-2 production, but due to specific metabolic effects, such as changes in fatty acid metabolism and cysteine/sulfur-containing amino acid metabolism^36,63^. Genes involved in cysteine synthesis were upregulated in *B*. *breve* UCC2003 under shock with 0.5% (w/v) porcine bile. LuxS is responsible for bifidobacterial synthesis of AI-2, yet bifidobacteria appear to lack an AI-2 quorum sensing system such as LuxP and/or LsrB, and we can therefore only speculate as to the mechanism by which AI-2 production is linked to bile resistance^43,64^. The *B*. *breve* UCC2003 EPS^−^ mutant has also been shown to be less resistant to 0.3% (w/v) porcine bile, while eliciting a reduced colonisation persistence in the GIT of mice^51^.

We also show that biofilm formation requires different macromolecular factors: the initial attachment phase of biofilm seems to be dependent on eDNA, EPS and protein interactions, though eDNA does not appear to be as important in the mature biofilm structure. A limitation of our study is that we could not distinguish if genes were important for initiation or maturation phases due to the screen being carried out in microtiter plates. More investigation is thus needed to dissect which genes are important for each of the phases of biofilm development and to discern if the importance of *luxS* in biofilm is due to AI-2 production or metabolic changes.

**Figure 7:**
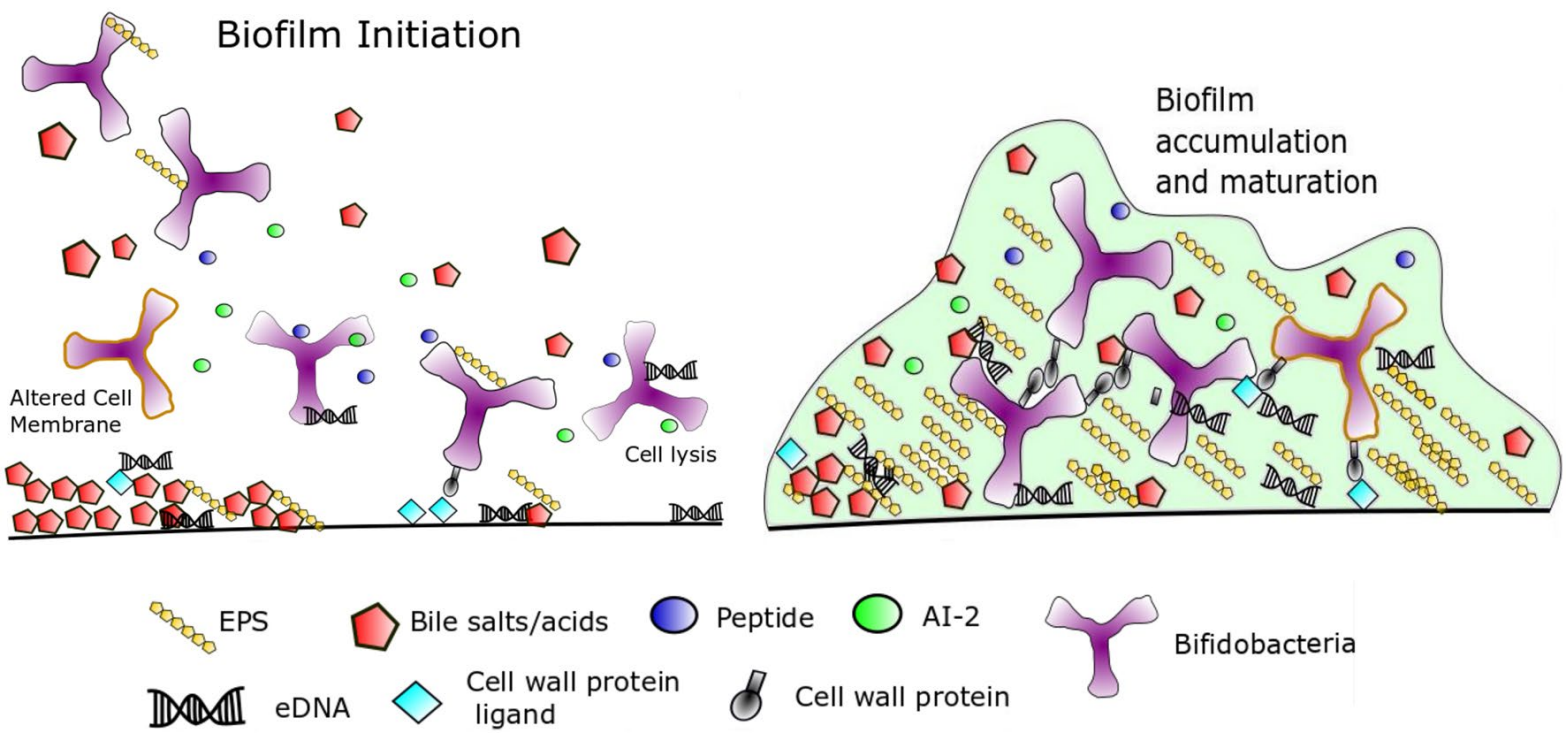
Model of biofilm formation by bifidobacteria induced by high concentrations of bile. See “Discussion” for details.

From our study we propose the following model of biofilm in bifidobacteria in response to high concentrations of bile based on our works findings and biofilm in the literature (Fig. 7). High concentrations of bile (0.5% and above) lyse bifidobacterial cells and may release intracellular signals such as AI-2 or oligopeptides to induce quorum sensing. Extracellular DNA released from lysed cells may also coat the surface and resulting additional electrostatic interactions that allow bifidobacteria to adhere. Bile increases hydrophobicity of the cell surface and allows initial attachment of bifidobacteria to the surface by increased hydrophobic interactions with the surface. Increased fatty acid biosynthesis may also alter cell surface membrane properties and LuxS may produce metabolic changes to also alter the cell membrane composition. Secretion of EPS and protein interactions may then allow firmer attachment and accumulation of cells. Maturation of the extracellular matrix of the biofilm involves further EPS secretion and protein interactions. When high concentrations of bile decrease, the biofilm may disperse and bifidobacterial cells are free to grow planktonically again. Future studies will be needed to test this model for accuracy, while additional studies are also needed to determine how important biofilm formation is for bifidobacterial gut colonisation and survival in specific parts of the GIT.

## Methods

### Bacterial strains, culture conditions, media

All bacterial strains used in this study are listed in Table 3. Bifidobacterial strains were routinely cultured in reinforced clostridial medium pH 6.8 (RCM, Oxoid Ltd., Basingstoke, Hampshire, United Kingdom) or reinforced clostridial agar (RCA, Oxoid Ltd.). RNAseq experiments were carried out using cultures that had been grown in filtered RCM (fRCM). All bifidobacterial strains were grown anaerobically in a modular atmosphere controlled system (Davidson and Hardy, Belfast, Ireland). Where required, media was supplemented with tetracycline (Tet, 10 µg ml^−1^) or porcine bile, 0.5% (w/v) or 1% (w/v) (Sigma- Aldrich, Steinheim, Germany). Individual bile salts were purchased from Sigma-Aldrich.

**Table 3:** Strains and plasmids used in this work.

Bacterial strain/plasmid	Features	References
*Bifidobacterium breve*		
UCC2003		^48^
UCC2003::Bbr_430	Insertional mutant in Bbr_430 gene of the EPS cluster	^51^
UCC2003-luxS	Insertion mutant in *luxS* – (Bbr_0541)	^43^
JCM 7017		
JCM 7019		
NCTC 11815		
*Bifidobacterium longum* subsp*. longum*		
NCIMB 8809		
CCUG 30698		
*Bifidobacterium longum* subsp*. infantis*		
ATTC 15697		
*Bifidobacterium dentium*		
DSM 20436		
*Bifidobacterium adolescentis*		
DSM 20083		
*Bifidobacterium pseudolongum*		
DSM 20438		

### Crystal violet biofilm assay

Overnight cultures of bifidobacteria (20 µl) were used to inoculate RCM supplemented with 0.5% (w/v) or 1% (w/v) porcine bile (Final volume 200 µl) in a 96 well microtiter plate. Biofilms were allowed to form for 24 h at 37 °C in anaerobic conditions and were then washed three times with deionised water to remove planktonic cells and left to dry for 1 h. The biofilms were stained with 1% crystal violet (100 µl) (Sigma-Aldrich) for 1 min and then washed three times with deionised water to remove excess crystal violet stain. Crystal violet stained biofilms were then solubilised with 5% (v/v) acetic acid (100 µl) (Sigma-Aldrich) and the absorbance read at a wave length of 570 nm.

### Screening of a mutant library for biofilm defective mutants

A previously described transposon mutant library of *B. breve* UCC2003^49,50^ was screened for mutants affected in their ability to from a biofilm. Individual transposon mutants were subcultured in RCM supplemented with tetracycline and RCM supplemented with 0.5% and 1% (w/v) porcine bile, to induce biofilm, for 24 h. Biofilm formation was stained as described above. Transposon mutants that exhibited reduced biofilm formation were then selected for further analysis. The precise location of the transposon in a given mutant was then identified by iPCR as previously described^49,50^ or by arbitrary primed PCR as previously described with modifications^65,66^. Round one of arbitrary PCR was carried out with primers Arb 1, Arb 6 and either TnTetR1 or TnTetL1 (Table 4). The successful PCR reaction was then used in a second PCR reaction (round 2) using primers Arb2 and either TnTetR2 or TnTetL2 (Table 4). PCR reactions from iPCR reactions and round 2 arbitrary PCR reactions were then sequenced (Eurofins, Ebersburg, Germany) to identify transposon insertion with primers pMod-Fw-seq and pMod-Rv-seq.

**Table 4:** Oligonucleotides used in this study.

Primer	Function	Sequence
iPCR-Fw	Forward primer for inverse PCR reaction	GCATACCGTACTGATCTG
iPCR-Rv	Reverse primer for inverse PCR reaction	CAATCATACCGGCTTCC
Arb6	Primer for arbitrary PCR	GGCCACGCGTCGACTAGTACNNNNNNNNNNACGCC
Arb2	Primer for arbitrary PCR	GGCCACGCGTCGACTTAGTTAC
Arb1	Primer for arbitrary PCR	GGCCACGCGTCGACTAGTTACNNNNNNNNNNGATAT
TnTetL1	Primer for arbitrary PCR	AAAACATGGTGTCCGTCCTC
TnTetR1	Primer for arbitrary PCR	TCGCTGGGATACTTGAACCA
TnTetL2	Primer for arbitrary PCR	GCTGTGGTGTTTGGTTGGAA
TnTetR2	Primer for arbitrary PCR	CTCTATGCGCCCCAGGAATA
pMod-Fw-seq	Forward sequencing primer based on transposon	GCCAACGACTACGCACTAGCC
pMod-Rv-seq	Reverse sequencing primer based on transposon	GAGCCAATATGCGAGAACACC

### DNA manipulations

DNA manipulations were carried out as previously described^67^. Oligonucleotides used in this study were synthesised by Eurofins (Ebersberg, Germany) and are listed in Table 4. Genomic isolations from *B*. *breve* UCC2003 were performed as described previously^68^. Inverse PCR and arbitrary PCR reactions to identify transposon insertion points, were carried out with the 2X Phusion Green HSII High Fidelity polymerase (Thermo-Scientific) and Q5 High Fidelity DNA polymerase (New England Biolabs), respectively. All PCR products were purified using the High Pure PCR Purification Kit (Roche). Restriction enzymes (Sigma Aldrich) and T4 DNA ligase (Promega) were used as stated in the manufacturer’s instructions.

### Transcriptomic analysis

An overnight culture of *B. breve* UCC2003 in RCM was used to inoculate (1% v/v) fRCM and this culture was grown until an O.D._600 nm_ between 0.5 and 0.6 was reached. The cells were then exposed to a bile shock by adding 0.5% (w/v; final concentration) porcine bile. Following 20 min bile exposure cells were harvested by centrifugation, while a culture in fRCM to which no porcine bile was added was also harvested as a control. RNA extraction was carried out as previously reported but with modifications^69^. In order to extract RNA, total RNA of each of the cultures was mixed with 800 µl of QIAzoL Lysis Reagent (Qiagen, UK) and pipetted in to a sterile tube with glass beads (Merck, Germany). Cells were lysed 2 min of stirring this mixture in a Precellys 24 homogenizer (Bertin instruments, France) with 2 min of static cooling; this step was repeated in triplicate. The lysed cells were centrifuged to remove cellular debris at 12,000 rpm for 15 min and the upper phase was collected. The RNA samples were purified using the RNAesy Mini Kit (Qiagen, UK) as per the manufacturer’s protocol. RNA concentration and purity were checked by a Picodrop microliter spectrophotometer (Picodrop, UK).

#### RNAseq analysis performed by NextSeq Illumina

RNAseq analysis was carried out as previously described with modifications^70^. A total of 2.5 µg of RNA was treated to remove ribosomal RNA by the Ribo-Zero Magnetic Kit (Illumina) for RNA sequencing, and the rRNA-depleted sample purified by ethanol precipitation. RNA was processed according to the manufacturer’s protocol. The yield of rRNA depletion was measured by a Tape station 2,200 (Agilent Technologies, USA). The construction of the whole transcriptome library was carried out using the TruSeq Stranded RNA LT Kit (Illumina). Samples were loaded into a NextSeq High Output v2 Kit Chemicals 150 cycles (Illumina) as per the technical support guide. The reads were depleted of adapters, quality filtered (with overall quality, quality window and length filters) and aligned to the *B*. *breve* UCC2003 genome.

#### Inhibition and dispersal assays

In order to study the factors involved in the initial steps of biofilm formation, an inhibition assay was performed as previously described^54^ with some modifications as follows. RCM supplemented with 0.5% (w/v) porcine bile was inoculated with 10% overnight *B*. *breve* UCC2003 wildtype (WT) strain and *B*. *breve* UCC2003::Bbr_430 (${\text{EPS}}^{-}$-negative phenotype)^51^. The RCM was also supplemented with 0.95 mg/ml proteinase K (Sigma Aldrich),10 U/µl DNase1 (Sigma Aldrich) or 4 mM sodium (meta) periodate (Sigma Aldrich). Cells were left to form biofilm anaerobically for 24 h at 37ºC, after which biofilm biomass was stained with crystal violet as described above. To investigate what mature biofilm biomass is composed of, biofilm was allowed to form for 24 h as for the inhibition assay and treated as previously described with modifications^71^. The planktonic phase was removed, and biofilms treated with 0.95 mg/ml of proteinase K in 20 mM Tris–HCl or 5 U/µl of DnaseI in 5 mM MgCl_2_ for a further 24 h at 37 °C anaerobically. Biofilms were then stained with crystal violet as stated above.

#### Viability assays

To access the viability of cultures after 24 h growth in bile, overnight cultures of bifidobacteria were inoculated as above for biofilm assays into either RCM supplemented with 0.5% (w/v) porcine bile or RCM only, as an untreated control, and incubated for 24 h. After this culture medium was diluted in PBS and spot plated onto RCA. Plates were incubated for 48 h anaerobically at 37ºC. Cultures were also grown in glass test tubes in the presence of 0.5% (w/v) bile and allowed to form biofilm for 24 h. Biofilm was then washed three times with sterile water and a pipette tip was used to scrape biofilm from the surface of the test tube. Biofilm was then restreaked on RCA supplemented with 0.05 (v/v) % cysteine-HCl (Sigma) and 0.3% (w/v) lactose (Sigma).

## Supplementary information

Supplementary figure 1
