# Supplementary material for: Bifidobacterial biofilm formation is a multifactorial adaptive phenomenon in response to bile exposure
Source: Sci Rep. 2020 Jul 14;10:11598. doi: 10.1038/s41598-020-68179-9 (PMC7360559; doi:10.1038/s41598-020-68179-9)
Supplement: Supplementary file 1 — Supplementary figure 1 [file 41598_2020_68179_MOESM1_ESM.pdf]

Supplementary Figure S1: Transposon mutants with reduced biofilm formation in *Bifidobacterium breve* UCC2003.

Caption:

Summary of the eleven transposon mutants affected in biofilm formation found during a screen of a transposon mutant library of *B*. *breve* UCC2003. Black triangles indicate the location of the transposon insertion.

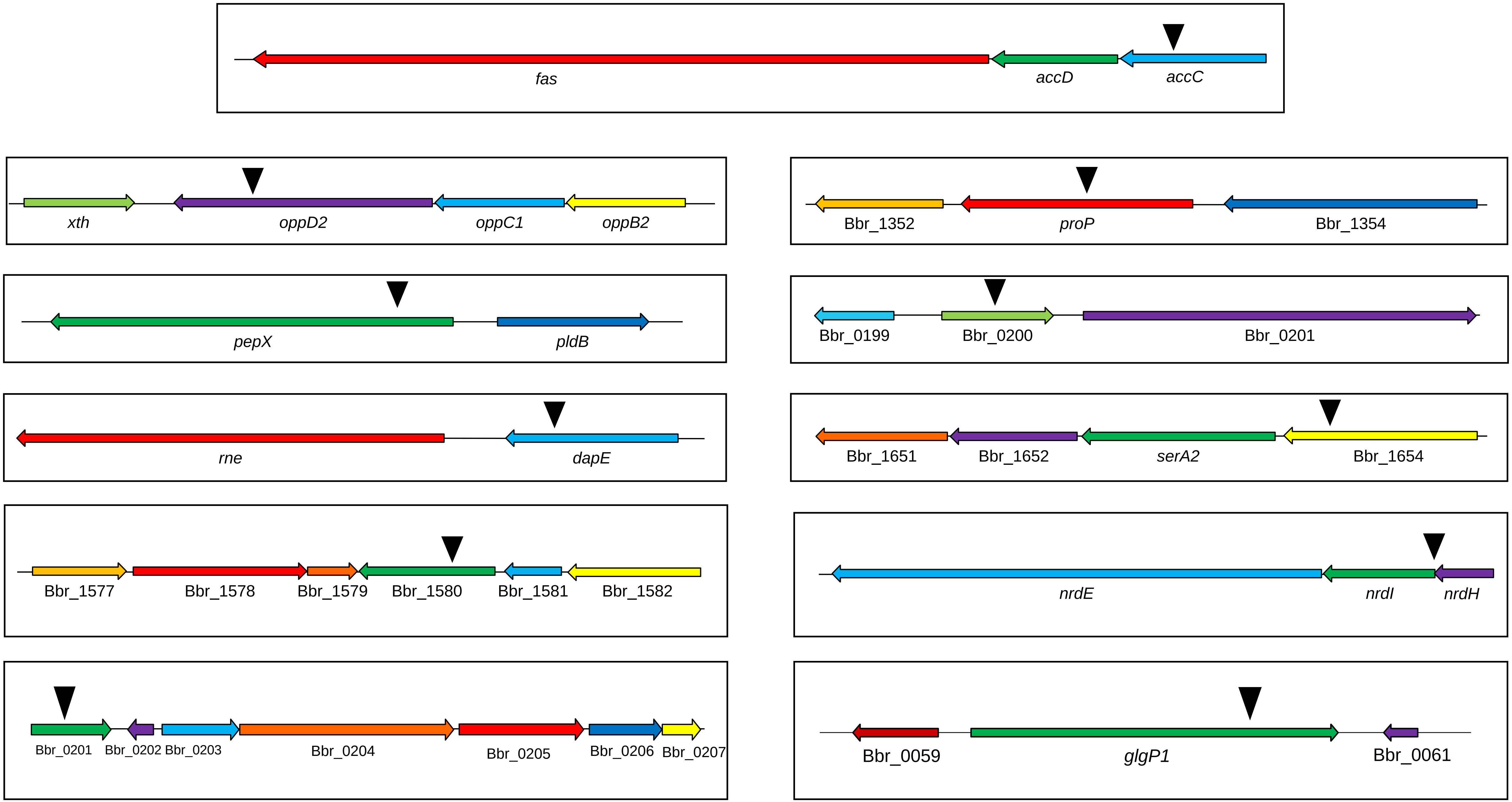
fas
accD
accC
xth
oppD2
oppC1
oppB2
Bbr_1352
proP
Bbr_1354
pepX
pldB
Bbr_0199
Bbr_0200
Bbr_0201
rne
dapE
Bbr_1651
Bbr_1652
serA2
Bbr_1654
Bbr_1577
Bbr_1578
Bbr_1579
Bbr_1580
Bbr_1581
Bbr_1582
nrdE
nrdI
nrdH
Bbr_0201
Bbr_0202
Bbr_0203
Bbr_0204
Bbr_0205
Bbr_0206
Bbr_0207
Bbr_0059
glgP1
Bbr_0061
